# Mechanisms by which Human Cells Bypass Damaged Bases during DNA Replication after Ultraviolet Irradiation

**DOI:** 10.1100/tsw.2002.348

**Published:** 2002-05-14

**Authors:** James E. Cleaver

**Affiliations:** UCSF Cancer Center, University of California, San Francisco, USA

**Keywords:** ultraviolet light, DNA repair, xeroderma pigmentosum, pyrimidine dimers, excision repair, squamous cell carcinoma, basal cell carcinoma, melanoma

## Abstract

The replication of damaged DNA involves cascading mechanisms of increasing complexity but decreasing accuracy. The most accurate mechanism uses low-fidelity DNA polymerases, Pol H and Pol I, which have active sites sufficiently large to accommodate a pyrimidine dimer. Replicative bypass of DNA damage by these polymerases produces an accurately replicated, newly synthesized strand. Pol H negative cells (XP-V cell lines) either adopt a proposed secondary bypass mechanism or a recombinational mode. The mechanism of the secondary bypass is unclear, but a number of experiments suggests roles for excision repair to remove damage ahead of replication forks, hRad6/18 proteolysis to clear the blocked forks, and the Rad17-RFC and 9-1-1 complexes to establish a new replication apparatus. This alternative pathway requires functional p53. In Pol H negative cells in which p53 is also inactive, the arrested fork fragments into DNA double strand breaks. Foci containing PCNA, Mre11/Rad50/Nbs1, and gamma-H2Ax can then be detected, along with chromosomal rearrangement and high frequencies of sister chromatid exchanges. The recruitment of recombination components to the arrested forks represents the ultimate failure of replication machinery to relieve the arrested state and bypass the damage. The resulting chromosomal instability in surviving cells will contribute to malignant transformation.

## INTRODUCTION

The study of DNA replication arrest in human cells after UV irradiation went through two major historical phases, driven by distinct technologies: the first phase employed alkaline sucrose gradients and the second employed the identification of molecular and enzymatic components through gene cloning, DNA sequencing, and functional assays.

The first phase occurred between approximately 1969 to 1979, during which a large body of work was done using alkaline sucrose gradients to determine changes in the sizes of nascent DNA after irradiation. By the end of the 1970s, the technology had been more or less exhausted. The existence of blocks to the progress of replication forks had been established[1,2]; bypass appeared to involve a replicative process rather than recombination[3,4]; gaps were apparent in the newly replicated DNA at what appeared to be pyrimidine dimer sites[1,5,6,7]; and replicative bypass appeared to result in the re-replication of up to 1 kb of the newly synthesised DNA[4]. One of the complementation groups of the disease xeroderma pigmentosum that exhibited greatly elevated skin carcinogenesis from UV exposure, the XP variant (XP-V), was shown to be deficient in bypass replication[8,9,10]. The replication arrest was greatly enhanced by incubation of UV irradiated cells in high concentrations of caffeine[6,11,12]. Ironically, an attempt to define the mechanism of replication arrest from UV damage, the process we called “postreplication repair,” in a publication of my own, seemed to presage a loss of interest and activity in the field for many years[3]!

The field then languished, despite suggestions from work associated with another genetic disease, ataxia telangiectasia (AT), that there were novel mechanisms to be discovered that prevented radiation damage from arresting DNA replication, as well as those enhancing the arrest. This remarkable observation of “radio-resistant DNA synthesis”[13] implied that the process of DNA replication, fork progression, and arrest was a complex cellular regulatory process that deserved focused attention.

The next phase, which is now moving remarkable rapidly, began in earnest with the discovery that a gene in yeast encoded a new DNA polymerase, *yRad30* (*pol eta*, *Pol H*)[14]. This became the starting point for discovery of a myriad of new DNA polymerases, especially a class-designated Y that had uniquely low replication fidelity[15]. A simplification of polymerase nomenclature has now replaced Greek letters with Roman capitals[16]. DNA photoproducts were known to be blocks to the replicative DNA polymerases, alpha, delta, and epsilon because they cannot accommodate large distortions such as DNA photoproducts or adducts in their active sites[17,18,19]. Replicative bypass of photoproducts was achieved instead by damage-specific polymerases with large substrate binding sites and relaxed substrate specificity[15,19]. These polymerases appear to require association with RFC, PCNA, and RPA to anchor them in the replication apparatus[20,21].

Three class Y genes have been identified in the mammalian genome, *POL H*, *I*, and *K*. *POL H* and *I* are paralogs, unique to mammalian cells, and only a single *POL H* gene is found in yeast[15]. Pol H has a high capacity for replicating many kinds of DNA lesions and preferentially inserts As opposite thymine-containing lesions[22]. Pol I has a poorer capacity for replication of UV damage, being only able to insert a base opposite the 3’ T of a [6-4] photoproduct and preferentially inserts Gs, suggesting a role in replication of C-containing photoproducts[23,24,25]. Pol K is very inefficient at replication of UV damage or abasic sites, but it can extend from a misincorporated G opposite the 3’ T of a photoproduct and other mismatched base pairs[26]. Pol Z and Pol K can therefore extend nascent DNA chains after Pol H or Pol I have inserted A or G opposite photoproducts, but since both can extend mismatched base pairs, they contribute positively to increased mutagenesis after UV irradiation[25,26,27].

Mutations in *POLH* are found in XP-V cells[28,29]. Many of the mutations cause severe truncations of the protein, and others prevent the enzyme from relocating to replication foci after UV irradiation[30,31,32]. The phenotype of XP-V cells, an enhanced arrest of DNA replication at pyrimidine dimer sites and increased mutagenesis[9,10,33,34], was therefore readily understood from the properties of Pol H and other damage-specific DNA polymerases.

The discovery of these polymerases at first solved many of the dilemmas associated with interpretation of older work on DNA replication after UV irradiation. With such solutions has come a range of new and interesting problems. For example, these polymerases have high error rates (1%) when assayed on undamaged DNA *in vitro*[22,35] that must be therefore controlled *in vivo*, otherwise the results would be catastrophic to the cell. One control mechanism is to exclude Pol H from replication sites until a cell is UV damaged[31,32]. But although translocation to arrested forks requires specific sequences in Pol H[32], the mechanism of translocation is unexplained. Questions remain for how XP-V cells bypass pyrimidine dimers in the absence of Pol H, which early recovery experiments suggest that they do at reduced rates[10]. The mechanism of caffeine-induced toxicity to UV damaged cells remains unexplained, despite many studies[12,36]. Although ATR is sensitive to caffeine, as are other PI3-kinases[37], the cellular responses are not easy to explain on the basis of such inhibition.

In the remaining sections I want to re-examine the consequences of replication arrest in the XP-V cells and ask whether older studies still suggest that there are additional processes yet to be understood in molecular terms.

## DNA Replication After UV Irradiation––Sizes of Nascent Strands

A major technique in early studies was to label cells with short pulses of [$^3$-H]-thymidine and then determine the sizes of nascent strands and the rates of new DNA replication. Subsequently the labeled DNA was chased into higher molecular weight DNA by growth in non-radioactive thymidine or bromodeoxyuridine (Fig. 1). Typical results led to the following conclusions:

1. Normal human cells suffered little interruption in replication and had a very efficient bypass mechanism.
2. Cells acquired an increased capacity to replicate DNA within a few hours of irradiation.
3. Excision repair defective cells showed a slower increase in DNA replication capacity, and XP-V cells had the least.
4. Maximum interruptions to DNA replication occurred by blocks at each pyrimidine dimer site.
5. Once DNA replication had been interrupted, normal and XP-V cells appeared to chase the short, labeled DNA into high molecular weight DNA at the same rates.

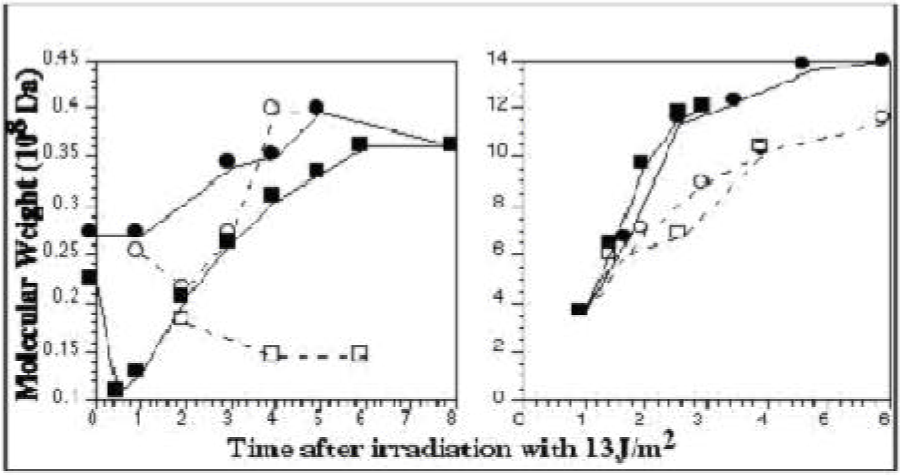

**FIGURE 1**. Replication of damaged DNA in normal and XP-V cells after 13 J.m$^{-2}$ UV. Left: cells were irradiated and labeled for 10 min at indicated times (hours) after irradiation, and the single strand molecular weights determined by alkaline sucrose gradients. Right: normal and XP-V cells in which the DNA was labeled at 1 h after irradiation and allowed to elongate and analyzed at indicated times. XP-V cells (closed squares, open squares); normal cells (closed circles, open circles). Cells in medium only (closed squares, closed circles); cells in 1 mM caffeine (open squares, open circles). Data reanalyzed from[3,10].

6. Production of high molecular weight DNA involved incorporation of new bases by replication into gaps of up to 1 kb in length.
7. Caffeine inhibited cells from acquiring increased capacity to replicate damaged DNA and slowed the assembly into high molecular weight DNA.
8. XP-V cells were most severely blocked in their initial attempts to replicate pyrimidine dimers in the presence of caffeine.

Subsequently, many discoveries have been made that are important for studies of DNA replication. One is the large field of kinase regulation of enzyme function by phosphorylation, some of which are kinases themselves, which can only be briefly addressed here[38,39]. These include ATM, ATR, CHK2, and DNA-PK. ATM is predominantly involved in the response of cells to ionizing radiation, although cells defective in ATM show radiation-resistant DNA synthesis at low UV doses[40]. ATR is involved mainly in replication arrest from hydroxyurea and UV light at higher doses and is caffeine-sensitive[37,38]. Very recently, another UV-specific kinase, homeodomain-interacting protein kinase-2 (HIPK2), which plays an important role in the UV response[41,42] has been identified. Notably, however, most early studies on DNA replication were done at low UV doses, below about 10–15 J.m$^{-2}$. Later biochemical studies have mostly been done at much higher, acutely toxic, doses of about 50 J.m$^{-2}$[41,42,43,44].

Several lines of evidence indicate that the toxic effects of DNA replication arrest from UV damage in XP-V cells are mediated by p53[39,45]. XPV cells that are p53 null (HPV16-E6 transformed) are very much more UV sensitive than fibroblasts with normal p53[46]. The UV sensitivity of SV40 transformed XP-V cells grown in caffeine is as great as p53 null XPV cells[47]. DNA replication was recognized early as occurring in visible foci where many individual replication forks were anchored. Various factors required for replication and repair accumulate at foci after irradiation and can be detected by immunofluorescence[48,49,50].

## DNA Replication Arrest in Light of the Class Y DNA Polymerases

The pronounced arrest of DNA replication in UV-irradiated XP-V cells and the associated increase in mutation rates[9,33,34] now are explicable regarding loss of the major pyrimidine dimer bypass polymerase, Pol H[28,29]. Loss of Pol H means that replication must occur through the action of other polymerases, Pol I, Pol K, Pol Z, and hREV1L and through recombination mechanisms that are less accurate. But the details are still unclear. The recovery of DNA replication in XP-V cells requires more study because it is not evident that we understand all that is involved (Fig. 1).

The initial arrest of DNA replication followed by the reassembly of short nascent DNA strands into high molecular weights (Fig. 1) fits neatly into a two-step model. Initial arrest and replication bypass involves Pol H and I. Subsequent extension from a mismatched 3' terminus involving the pyrimidine dimer on the parental strand, involves Pol Z and Pol K (Fig. 2)[25,26,51]. This model still does not, however, fully account for the older data and some of the newer information. Observations of DNA replication arrest and recovery better fit into a sequential, multi-step series of cellular attempts to negotiate damaged template bases. Each attempt either succeeds or hands on the arrested fork to the next set of molecular actors ending in broken forks and double-strand DNA breakage[49,52]. This is illustrated as a series of first, second, and third “passes” around DNA photoproducts (Fig. 3).

### *First Pass: Two Steps in the Bypass Mechanism of Replication*

In normal cells, with a complete suite of damage-specific polymerases and normal p53, UV damage in front of an advancing replication fork causes a temporary block to Pol D advancement. Pol D is displaced and Pol H and I are then recruited by means of their relocation signals[31,32] and PCNA

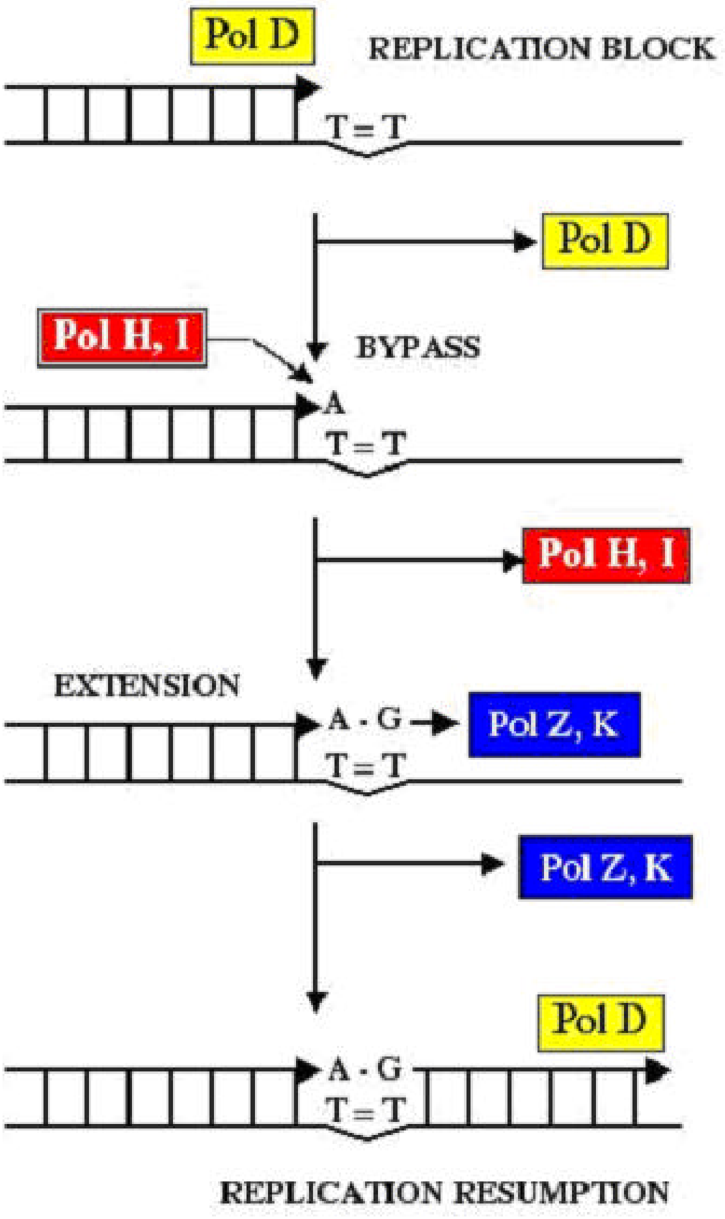

**FIGURE 2.** Sequential action of Pol H or I, followed by Pol Z or K, in bypassing UV photoproducts during DNA replication arrest. The initial base insertion opposite photoproducts can be carried out by the low fidelity polymerases Pol H and I. Extension from mismatched or poorly hydrogen-bonded base pairs can occur by the action of Pol Z or K. Once the photoproduct and the mismatched region have been negotiated, normal fork progression can resume with Pol D.

binding sites[21,53]. Single-strand binding protein (RPA) is phosphorylated during replication arrest and may also play a role[54,55,56]. These polymerases extend the nascent DNA chain by one or two bases across from the photoproducts, and there may be an additional role for editing by separate exonucleases. This results in the addition of As by Pol H across from thymine-containing photoproducts facilitating accurate replication of a T-T pyrimidine dimer[22]. The addition of Gs by Pol I, across from cytosine-containing photoproducts can also result in accurate replication of a C-containing pyrimidine dimer[57]. These steps will advance the replication fork to incorporate bases opposite the photoproduct but with potential mismatches at the 3’ terminus. Mismatched base pairs can then be extended by the action of Pol Z or K[25,26]. Presumably, a final handoff to the normal replicative polymerase, Pol D, will allow replication to resume after bypass is complete.

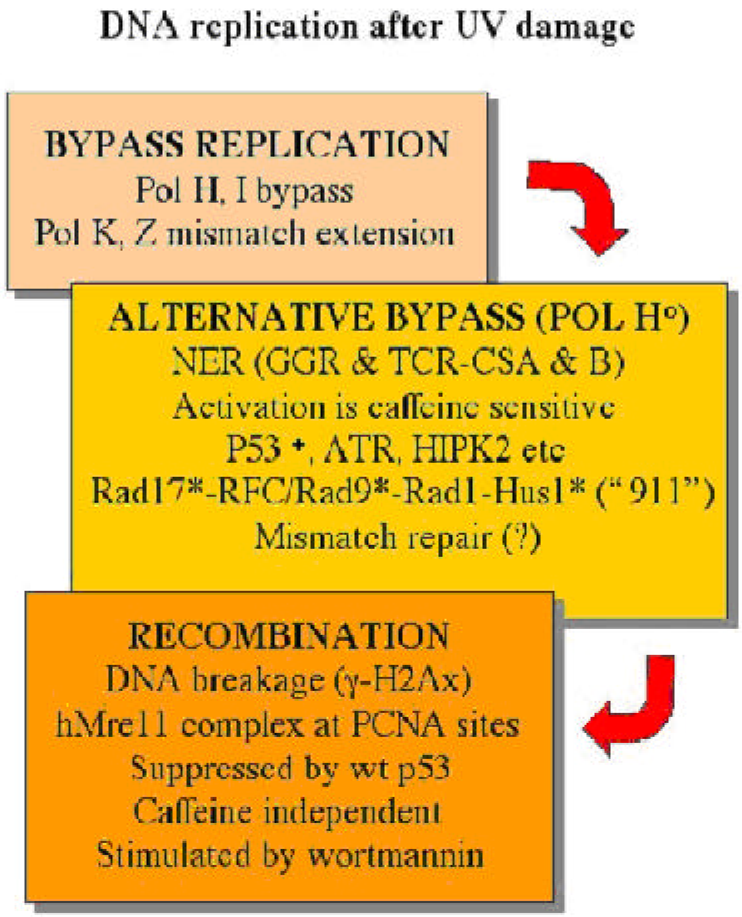

**FIGURE 3**. Three alternative sequential mechanisms that may occur during DNA replication arrest. Replicative bypass, using Pol H, I, K, and Z, is the preferred mode in normal cells. Mre11-mediated recombination occurs in cells deficient in Pol H and p53. An alternative mode involving excision repair, kinase activation and other factors is suggested on the basis of a series of experiments, and may be involved in Pol H deficient, XP-V cells with normal p53; phosphorylation sites shown by *.

Work with XP-V cells and plasmid-borne target genes shows that the low-fidelity polymerases provide the structural flexibility to bypass DNA photoproducts accurately, and in their absence mutation rates from UV damage increases[33]. Conversely, the down-regulation of Pol Z or the terminal dCMP-transferase hRev1 (REV1L) by antisense results in reduced mutation rates[27,58].

Older work on recovery of DNA replication and of bypass (Fig. 1) indicates that XP-V cells still retain the capacity to recover replication capacity and can bypass photoproducts with reduced fidelity. In part, this may be caused by Pol I function in Pol H defective cells, but there may be additional processes. In p53 defective, transformed cells, this alternative pathway involves recombination, to be discussed later, but in p53 wild-type cells a route can be hypothesized that does not involve recombination.

## *Second Pass: Activation of the 9-1-1 & RFC-Rad17 Complexes by ATR Kinase*

How do arrested replication forks bypass photoproducts in Pol H negative cells? This may not be a simple process. Bypass may involve excision of pyrimidine dimers by NER[59]. But excision may be unlikely for dimers already in blocked forks that are partially single stranded. In general excision is slower than the rate of recovery of replication capacity (Fig. 1). There may consequently be a role for other factors known, from yeast and human cells, to be players in the damage response to UV in the “post-replication” pathways or epistasis groups. Most notably are the hRad17-RFC complex and the hRad9-hRad1-Hus1 trimer (9-1-1 complex)[60]. These complexes appear to be damage-specific checkpoint gene products that resemble the RFC loading factor and the PCNA polymerase clamp and are also involved in binding and signal transduction associated with double strand breaks. Ubiquitination by hRad6/18 may also be required to clear blocked polymerases and other factors from the forks, which appears to act as a central determinant of postreplication repair[61].

Another question is whether components of the mismatch repair system could play a role. These components are found associated with other related factors in the replication complex[50,62]. Mismatch repair can correct replication errors; the distortion caused by mismatches and pyrimidine dimers are superficially similar, and the regions resynthesized during mismatch repair and completion of postreplication repair gaps are equally large. It would therefore not be surprising to find a role for mismatch repair in recovery of DNA replication from UV damage, just as it plays a role in strand selection during transcription-coupled excision repair[63,64,65]. The transcription coupling factors, CSA and CSB, also may be involved because Cockayne syndrome cells show a reduced capacity to recover DNA replication after UV as well as their reduced recovery of RNA transcription[66,67]. Many of these factors require phosphorylation, especially p53, hRad9, Hus1, and hRad17, suggesting that activation of ATR and other kinases by UV during replication arrest is important in stimulating this recovery process[38,39,60].

### *Third Pass: Fork Collapse and Mre11-Mediated Recombination*

Exchanges between sister chromatids (SCEs) are greatly increased in Pol H-negative transformed cells at very low doses of about 1 $J.m^{-2}$, indicating that UV can stimulate effective recombination[46]. In addition, foci are detectable containing PCNA, the Mre11 complex, and gamma-H2Ax[49,52]. The association with PCNA indicated that these foci were at replication forks. The accumulation of Mre11 and gamma-H2Ax was an indication that the structures at the forks resembled loops and breaks that are known substrates of Mre11, which develop in the absence of Pol H and of p53[68]. Coimmunoprecipitation with an antibody to Mre11 brought down the other components of the trimer, hRad50 and Nbs1, but not PCNA from UV-irradiated XP-V cells (Laposa and Cleaver, 2002, unpublished observations).. Therefore, although these proteins are physically close, they do not appear to interact directly but rather are linked by their proximity on regions of a common DNA structure.

The precise role of p53 that has been eliminated by transformation in these studies is unclear, but recombination may be suppressed by direct protein-protein interaction, or masking of DNA binding sites by p53’s propensity for nonspecific DNA binding. Inactivation of p53 either by p53-largeT binding, or E6 ubiquitination and destruction, allows Mre11 and gamma-H2Ax foci to accumulate[47]. Homologous recombination is suppressed by direct interaction between Rad51 with p53[69,70,71]. Surprisingly, we found that Rad51 and Brca1 formed foci in XP-V cells in response to UV damage, which were distant from the replication forks and from PCNA. These foci were similar in normal and XP-V cells after both UV and hydroxyurea treatment (Scully and Cleaver unpublished observations). Therefore the events at the replication fork recruit components of the double strand break repair, Mre11 complex and gamma-H2Ax, that act before Rad51-dependent homologous recombination, and which are themselves also suppressed by p53[47]. Another site of action of p53 may be its binding to RPA that is especially important to DNA replication after UV damage because of the lengthy single stranded regions exposed during the arrest[72,73].

The recruitment of recombination components to the arrested forks represents the ultimate failure of replication machinery to recover from the arrested state, and this will cause chromosomal instability in surviving cells that can contribute to malignant transformation.

## ACKNOWLEDGMENTS

The work described here was supported by the National Institutes of Environmental Health Sciences Grant 1 RO1 ES 8061 (to J.E.C.); the Ellison Senior Scholar Award (to J.E.C). I am also very grateful to the XP Society, Poughkeepsie, NY, and the Kevin O'Brien Foundation, Wilton, CT, for their continued support and encouragement.

## References

1. Menighini, R. (1976) Gaps in DNA synthesised by ultraviolet light-irradiated WI38 human cells. *Biochim. Biophys. Acta* **425**, 419–427.
2. Park, S.D. and Cleaver, J.E. (1979) Recovery of DNA synthesis after ultraviolet irradiation of xeroderma pigmentosum cells depends on excision repair and is blocked by caffeine. *Nucleic Acids Res*. **6**, 1151–1159.
3. Park, S.D. and Cleaver, J.E. (1979) Postreplication repair: questions of its definition and possible alterations in xeroderma pigmentosum cell strains. *Proc. Natl. Acad. Sci. U. S. A.* **76**, 3927–3931.
4. Lehmann, A.R. (1972) Postreplication repair of DNA in ultraviolet-irradiated mammalian cells. *J. Mol. Biol.* **66**, 319–337.
5. Clarkson, J.M. and Hewitt, R.R. (1976) Significance of dimers to the size of newly synthesized DNA in UV-irradiated Chinses hamster ovary cells. *Biophys. J.* **16**, 1155–1164.
6. Lehmann, A.R. (1979) The relationship between pyrimidine dimers and replicating DNA in ultraviolet light-irradiated human fibroblasts. *Nucleic Acids Res.* **7**, 1901–1912.
7. Menighini, R. and Hanawalt, P.C. (1976) T4-endonuclease V-sensitive sites in DNA from ultraviolet-irradiated human cells. *Biochim. Biophys. Acta* **425**, 428–437.
8. Cleaver, J.E. (1972) Xeroderma pigmentosum: variants with normal DNA repair and normal sensitivity to ultraviolet light. *J. Invest. Dermatol*. **58**, 124–128.
9. Lehmann, A.R. et al. (1975) Xeroderma pigmentosum cells with normal levels of excision repair have a defect on DNA synthesis after UV-irradiation. *Proc. Natl. Acad. Sci. U. S. A.* **72**, 219–235.
10. Cleaver, J.E., Thomas, G.H., and Park, S.D. (1979) Xeroderma pigmentosum variants have a slow recovery of DNA synthesis after irradiation with ultraviolet light. *Biochim. Biophys. Acta* **564**, 122–131.
11. Cleaver, J.E. and Thomas, G.H. (1969) Single strand interruptions in DNA and the effects of caffeine in Chinese hamster cells irradiated with ultraviolet light. *Biochem. Biophys. Res. Commun.* **36**, 203–208.
12. Domon, M. and Rauth, A.M. (1969) Effects of caffeine on ultraviolet irradiated mouse L cells. *Radiat. Res.* **39**, 207–221.
13. Painter, R.B. (1981) Radioresistant DNA synthesis: an intrinsic feature of ataxia telangiectasia. *Mutat. Res.* **84**, 183–190.
14. Johnson, R.E., Prakash, S., and Prakash, L. (1999) Efficient bypass of a thymine-thymine dimer by yeast DNA polymerase eta. *Science* **283**, 1001–1004.
15. Ohmori, H. et al. (2001) The Y-Family of DNA Polymerases. *Mol. Cell*. **8**, 7-8.
16. Burgers, P.M. et al. (2001) Eukaryotic DNA polymerases: proposal for a revised nomenclature. *J. Biol. Chem.* **276**, 43487–43490.
17. Steitz, T.A. (1999) DNA polymerases: structural diversity and common mechanisms. *J. Biol. Chem.* **274**, 17395–17398.
18. Brash, D.E. et al. (1991) A role for sunlight in skin cancer: UV-induced p53 mutations in squamous cell carcinoma. *Proc. Natl. Acad. Sci. U. S. A.* **88**, 10124–10128.
19. Trincao, J. et al. (2001) Structure of the catalytic core of S. cerevisiae DNA polymerase h: implications for translesion synthesis. *Mol. Cell Biol.* **8**, 417–426.
20. Haracska, L. et al. (2002) Stimulation of DNA synthesis activity of human DNA polymerase kappa by PCNA. *Mol. Cell Biol.* **22**, 784–791.
21. Haracska, L. et al. (2001) Interaction with PCNA is essential for yeast DNA polymerase h function. *Mol. Cell Biol.* **8**, 407–415.
22. Johnson, R.E. et al. (2000) Fidelity of human DNA polymerase h. *J. Biol. Chem*. **275**, 7447–7450.
23. Tissier, A. et al. (2000) Pol iota, a remarkable error-prone human DNA polymerase. *Genes Dev.* **14**, 1642–1650.
24. Tissier, A. et al. (2000) Misinsertion and bypass of thymine-thymine dimers by human DNA polymerase iota. *EMBO J.* **19**, 5259–5266.
25. Johnson, R.E. et al. (2000) Eukaryotic polymerases i and z act sequentially to bypass DNA lesions. *Nature* **406**, 1015–1019.
26. Washington, M.T. et al. (2002) Human *DINB1*-encoded DNA polymerase k is a promiscuous extender of mispaired promer termini. *Proc. Natl. Acad. Sci. U. S. A.* **99**, 1910–1914.

27. Gibbs, P.E. et al. (1998) A human homolog of the Saccharomyces cerevisiae REV3 gene, which encodes the catalytic subunit of DNA polymerase zeta. *Proc. Natl. Acad. Sci. U. S. A.* **95**, 6876–6880.
28. Johnson, R.E. et al. (1999) *hRAD30* mutations in the variant form of xeroderma pigmentosum. *Science* **264**, 263–265.
29. Masutani, C. et al. (1999) The *XPV* (xeroderma pigmentosum variant) gene encodes human DNA polymerase h. *Nature* **399**, 700–704.
30. Broughton, B.C. et al. (2002) Molecular analysis of mutations in DNA polymerase eta in xeroderma pigmentosum-variant patients. *Proc. Natl. Acad. Sci. U. S. A.* **99**, 815–820.
31. Thakur, M. et al. (2001) DNA polymerase h undergoes alternative splicing, protects against UV sensitivity and apoptosis, and suppresses Mre11-dependent recombination. *Genes Chrom. Cancer* **32**, 222–235.
32. Kannouche, P. et al. (2001) Domain structure, localization, and function of DNA polymerase eta, defective in xeroderma pigmentosum variant cells. *Genes Dev*. **15**, 158–172.
33. Wang, Y.C., Maher, V.M., and McCormick, J.J. (1991) Xeroderma pigmentosum variant cells are less likely than normal cells to incorporate dAMP opposite photoproducts during replication of UV-irradiated plasmids. *Proc. Natl. Acad. Sci. U. S. A.,* **88**, 7810–7814.
34. Wang, Y.C. et al. (1993) Evidence from mutation spectra that the UV hypermutability of xeroderma pigmentosum variant cells reflects abnormal error-prone replication on a template containing photoproducts. *Mol. Cell. Biol.* **13**, 4276–4283.
35. Matsuda, T. et al. (2000) Low fidelity DNA synthesis by human DNA polymerase eta. *Nature* **404**, 1011–1013.
36. Cleaver, J.E. (1989) Caffeine toxicity is inversely related to DNA repair in simian virus-40 transformed xeroderma pigmentosum cells irradiated with ultraviolet light. *Teratog. Carcinog. Mutagen.* **9**, 147–155.
37. Sarkaria, J.N. et al. (1999) Inhibition of ATM and ATR kinase activities by the radiosensitizing agent, caffeine. *Cancer Res.* **59**, 4375–4382.
38. Shiloh, Y. (2001) ATM and ATR: networking cellular responses to DNA damage. *Curr. Opin. Genet. Dev.* **11**, 71–77.
39. Ljungman, M. (2000) Dial 9-1-1 for p53: mechanisms of p53 activation by cellular stress. *Neoplasia* **2**, 208–225.
40. Painter, R.B. (1985) Inhibition and recovery of DNA synthesis in human cells after exposure to ultraviolet light. *Mutat. Res.* **145**, 63–69.
41. Hofmann, T.G. et al. (2002) Regulation of p53 activity by its interaction with homeodomain-interacting protein kinase-2. *Nat. Cell Biol.* **4**, 1–10.
42. D'Orazi, G. et al. (2002) Homeodomain-interacting protein kinase-2 phosphorylates p53 at Ser 46 and mediates apoptosis. *Nat. Cell Biol.* **4**, 11–19.
43. Chehab, N.H. et al. (1999) Phosphorylation of ser-20 mediates stabilization of human p53 in response to DNA damage. *Proc. Natl. Acad. Sci. U. S. A.* **96**, 13777–13782.
44. Chao, C. et al. (2000) Phosphorylation of murine p53 at ser-18 regulates the p53 responses to DNA damage. *Proc. Natl. Acad. Sci. U. S. A.* **97**, 11936–11941.
45. Giaccia, A.J. and Kastan, M.B. (1998) The complexity of p53 modulation: emerging patterns from divergent signals. *Genes Dev.* **12**, 2973–2983.
46. Cleaver, J.E. et al. (1999) Increased UV sensitivity and chromosomal instability related to p53 function in the xeroderma pigmentosum variant. *Cancer Res.* **59**, 1102–1108.
47. Cleaver, J.E. et al. (2001) Polymerase h and p53 jointly regulate cell survival, apoptosis and Mre11 recombination during S phase checkpoint arrest after UV irradiation. *DNA Repair* **3**, 1–17.
48. Scully, R. et al. (1997) Dynamic changes of BRCA1 subnuclear location and phosphorylation state are initiated by DNA damage. *Cell* **90**, 425–435.
49. Limoli, C.L. et al. (2000) Polymerase h deficiency in the XP variant uncovers an overlap between the S phase checkpoint and double strand break repair. *Proc. Natl. Acad. Sci. U. S. A.* **97**, 7939–7946.
50. Wang, Y. et al. (2000) BASC, a super complex of BRCA1-associated proteins involved in the recognition and repair of aberrant DNA structures. *Genes Dev.* **14**, 927–939.
51. Woodgate, R. (2001) Evolution of the two-step model for UV-mutagenesis. *Mutat. Res.* **485**, 83–92.
52. Limoli, C.L. et al. (2002) UV-induced replication arrest in the xeroderma pigmentosum variant leads to double strand breaks, g-H2Ax formation, and Mre11 relocalization. *Proc. Natl. Acad. Sci. U. S. A.* **99**, 233–238.
53. Haracska, L. et al. (2001) Physical and functional interactions of human DNA polymerase eta with PCNA. *Mol. Cell Biol.* **21**, 7199–7206.
54. Zernik-Kobak, M. et al. (1997) Sites of UV-induced phosphorylation of the p34 subunit of replication protein A from HeLa cells. *J. Biol. Chem*. **272**, 23896–23904.
55. Rodrigo, G. et al. (2000) DNA replication but not nucleotide excision repair is required for UVC-induced replication protein A phosphorylation in mammalian cells. *Mol. Cell Biol.* **20**, 2696–2705.
56. Oakley, G.G. et al. (2001) UV-induced hyperphosphorylation of replication protein A depends on DNA replication and expression of ATM protein. *Mol. Cell Biol.* **12**, 1199–1213.
57. Vaisman, A. and Woodgate, R. (2001) Unique misinsertion specificity of pol iota may decrease the mutagenic potential of deaminated cytosines. *EMBO J.* **20**, 6520–6529.

58. Gibbs, P.E. et al. (2000) The function of the human homolog of Saccharomyces cerevisiae REV1 is required for mutagenesis induced by UV light. *Proc. Natl. Acad. Sci. U. S. A.* **97**, 4186–4191.
59. Cleaver, J.E. et al. (1983) Replicon size and excision repair as factors in the inhibition and recovery of DNA synthesis from ultraviolet damage. *Biochim. Biophys. Acta* **739**, 207–215.
60. Lindsey-Boltz, L.A. et al. (2001) Purification and characterization of human DNA damage checkpoint Rad complexes. *Proc. Natl. Acad. Sci. U. S. A.* **98**, 11236–11241.
61. Xiao, W. et al. (2000) The Saccharomyces cerevisiae RAD6 group is composed of an error-prone and two error-free postreplication repair pathways. *Genetics* **155**, 1633–1641.
62. Simbulan-Rosenthal, C.M. et al. (1996) The expression of poly(ADP-ribose) polymerase during differentiation-linked DNA replication reveals that it is a component of the multiprotein DNA replication complex. *Biochemistry* **35**, 11622-11633.
63. Leadon, S.A. and Avrutskaya, A.V. (1997) Differential involvement of the human mismatch repair proteins, hMLH1 and hMSH2, in transcription-coupled repair. *Cancer Res*. **57**, 3784–3791.
64. Leadon, S.A. and Avrutskaya, A.V. (1998) Requirement for DNA mismatch repair proteins in the transcription-coupled repair of thymine glycols in Saccharomyces cerevisiae. *Mutat. Res.* **407**, 177–187.
65. Mellon, I. et al. (1996) Transcription-coupled repair deficiency and mutations in mismatch repair genes. *Science* **272**, 557–560.
66. Cleaver, J.E. (1982) Normal reconstruction of DNA supercoiling and chromatin structure in Cockayne syndrome cells during repair of damage from ultraviolet leight. *Am. J. Hum. Genet.* **34**, 566–575.
67. Lehmann, A.R., Kirk-Bell, S., and Mayne, L. (1979) Abnormal kinetics of DNA synthesis in ultraviolet light-irradiated cells from patients with Cockayne syndrome. *Cancer Res.* **39**, 4237–4241.
68. Lobachev, K.L., Gordenin, D.A., and Resnick, M.A. (2002) The Mre11 complex is required for repair of hairpin-capped double-strand breaks and prevention of chromosome rearrangements. *Cell* **108**, 183–193.
69. Buchhop, S. et al. (1997) Interaction of p53 with the human rad51 protein. *Nucleic Acids Res.* **25**, 3868–3874.
70. Mekeel, K.L. et al. (1997) Inactivation of p53 results in high rates of homologous recombination. *Oncogene* **14**, 1847–1857.
71. Willers, H. et al. (2000) Loss of wild-type p53 function is responsible for upregulated homologous recombination in immortal rodent fibroblasts. *Int. J. Radiat. Biol.* **76**, 1055–1062.
72. Dutta, A. et al. (1993) Inhibition of DNA replication factor RPA by p53. *Nature* **365**, 79–82.
73. Cordeiro-stone, M. et al. (1999) Analysis of DNA replication forks encountering a pyrimidine dimer in the template to the leading strand. *J. Mol. Biol.* **289**, 1207–1218.

## BIOSKETCH

**James E. Cleaver,** Ph.D., is Professor of Dermatology & Pharmaceutical Chemistry, and Leader, Cutaneous Oncology Program, Auerback Melanoma Laboratory, UCSF Cancer Center, University of California, San Francisco. He holds a B.A. in Natural Sciences (1961) and a Ph.D. in Medicine (1964) from Cambridge University. Dr. Cleaver's major research interest is in the mechanisms that maintain the stability of DNA in human cells, especially in the hereditary human skin cancers of xeroderma pigmentosum, and the associated neurodegeneration seen in many patients. Our emphasis is on nucleotide excision repair and mechanisms of recombination and apoptosis that are triggered by arrested DNA replication forks and DNA double strand breaks. The XP variant cells that lack a polymerase, Pol H, show premature arrest of DNA replication forks during the S phase in UV damaged cells, and increased UV-induced mutagenesis, and represent a defect associated with replication fidelity. The phenotype of XP variant cells can be significantly altered by transformation using SV40 or HPV16, indicating an interaction between arrested replication forks and p53, which influences genomic stability after UV irradiation. We also discovered that arrested DNA replication forks develop into DNA double strand breaks that stimulate recombination. Dr. Cleaver has received numerous honors and awards, including election to the National Academy of Sciences, 1999; Roll of Honor, International Union for Research on Cancer, Geneva, Switzerland, 1997; Senior Investigator award, American Society for Photobiology, 1995; Luigi Provasoli Award from American Society of Phycology, 1991; Alexander Hollaender Distinguished Postdoctoral Fellowship (D.L. Mitchell), 1989-1990; Lila Gruber Cancer Award from American Academy of Dermatology, 1976; Annual Award for Outstanding Research from the Radiation Research Society, 1973; and Gold Merit Award, Annual Meeting of the American Academy of Dermatology, 1968.
